# Cognitive Impairments and blood-brain Barrier Damage in a Mouse Model of Chronic Cerebral Hypoperfusion

**DOI:** 10.1007/s11064-022-03799-3

**Published:** 2022-10-29

**Authors:** Lu Yang, Jiangman Song, Di Nan, You Wan, Huailian Guo

**Affiliations:** 1 Department of Neurology, People’s Hospital, Peking University, Beijing, China; 2 Key Laboratory for Neuroscience, National Health and Family Planning Commission, Ministry of Education, Peking University, Beijing, China

**Keywords:** Blood-brain barrier, Chronic cerebral hypoperfusion, Cognition, Evans blue, Tight junction

## Abstract

Chronic cerebral hypoperfusion (CCH) is commonly involved in various brain diseases. Tight junction proteins (TJs) are key components constituting the anatomical substrate of the blood-brain barrier (BBB). Changes in cognitive function and BBB after CCH and their relationship need further exploration. To investigate the effect of CCH on cognition and BBB, we developed a bilateral common carotid artery stenosis (BCAS) model in *Tie2-GFP* mice. Mice manifested cognitive impairments accompanied with increased microglia after the BCAS operation. BCAS mice also exhibited increased BBB permeability at all time points set from D1 to D42. Furthermore, BCAS mice showed reduced expression of TJs 42 d after the operation. In addition, correct entrances of mice in radial arm maze test had a moderate negative correlation with EB extravasation. Our data suggested that BCAS could lead to cognitive deficits, microglia increase and BBB dysfunction characterized by increased BBB permeability and reduced TJs expression level. BBB permeability may be involved in the cognitive impairments induced by CCH.

## Introduction

Vascular dementia is the second common cause of dementia following AD [1]. CCH resulting from many vascular risk factors, for instance, hypertension and arthrosclerosis [2], is a common cause of vascular dementia in the elderly. CCH has been demonstrated to cause various cognitive deficits [3]. Additionally, CCH in AD transgenic mice suffered more severe cognitive impairments than that without hypoperfusion [4]. CCH also promotes Amyloid-β pathogenesis [5], and Amyloid-β protein deposition was found in mice subjected to BCAS [6], indicating that CCH could also trigger Alzheimer’s disease.

Various animal models have been established to reveal the pathophysiologic mechanism of CCH. In mice, commonly used models include unilateral common carotid artery occlusion, unilateral internal carotid artery occlusion [7], modified common carotid artery occlusion with unilateral common carotid artery permanent ligation and the contralateral transient ligation [8], or BCAS, which was firstly established by Shibata M in 2004 [9] and widely used recently. With certain inner diameter microcoils twined on bilateral common carotid arteries, the BCAS model provides good reproducibility of the CCH and resultant white matter lesions [10].

BBB as part of the neurovascular unit, is composed of vascular endothelial cells, pericytes, extracellular matrix and astrocytes and strictly controls the passage of molecules between blood and brain parenchyma. BBB was proved to be a natural barrier preventing harmful substances in the blood from entering into the brain. TJs are the main composition of endothelial cells regulating transcellular transport, forming a network spanning the cell circumferentially [11]. When vascular events occurred, various components of the BBB changed accordingly, leading to tissue damage and dysfunction, and may ultimately resulted in cognitive impairment [12]. BBB integrity was also compromised in AD patients and AD transgenic mice brains, indicating that BBB might also be involved in AD cognitive deficits, though the underlying relationship remains elusive [13]. The role of blood-brain barrier in cognitive changes after CCH needs further exploration.

Here, in the current study, we developed a BCAS model by narrowing the bilateral common carotid arteries with 0.18 mm inner diameter microcoils. Cognitive functions, microglia quantity, BBB permeability as well as the expression of TJs were then examined. Correlation analysis was performed to assess the relationship between mice cognitive performance and BBB permeability.

## Materials and methods

### Animal

*Tie2-GFP* transgenic mice *[STOCK Tg (TIE2GFP) 287Sato/J]* of 8–10 weeks (body weight 25 to 30 g) were used in the experiment. The transgenic mice express green fluorescent protein (GFP) specifically in endothelial cells allowing visualization of blood vessels under microscope [14]. The mice were housed in cages under a 12-hour light/dark cycle (lights on at 6:00 PM) with standard dry food pellets and water available ad libitum. All procedures were performed in accordance with the National Institutes of Health guide for the care and use of laboratory animals and approved by the Institutional Animal Care and Use Committee of Peking University People’s Hospital.

### Surgical procedure of bilateral common carotid artery stenosis

Animals were subjected to sham or BCAS operation randomly. Mice were anesthetized with sodium pentobarbital (1%, 5 ml/kg) by intraperitoneal injection. A midline cervical incision was made to expose and separate bilateral common carotid arteries from their sheaths under an operating microscope, two silk sutures were put around the distal and proximal parts of the right common carotid artery, the artery was gently lifted by the suture and then a microcoil with an internal diameter of 0.18 mm (Sawane Spring, Japan) was twined by rotating it around the proximal part of the carotid bifurcation, another microcoil of the same size was twined around the left common carotid artery 30 min later, as previously described [15]. Mice in sham group underwent the same procedure without microcoils on the vessels. Fine sutures were conducted to close the incision, mice were returned to the animal holding area after they appear normal. Body temperature was maintained by a small-animal warmer/thermometer system (BWT-100 A, Bio Research Center, Japan) during the operation.

### Radial arm maze test

Radial arm maze test began on D28 after the operation as described previously [15, 16]. The maze consists of eight arms (8 cm × 35 cm) that radiated from central starting zone. The test lasted 14 days during which body weight of the mice was monitored every day. Mice body weight was controlled to 85–90% of their basal value by appropriate amount of cheese-adapted diet within 7 days and were maintained till the end of the test. Mice that did not adapt well to the diet and lost weight too quickly were removed. On the first 3 d, mice were put in the radial arm maze for 5 min per day to adapt to the environment. On D4 and D5, small pieces of cheese were evenly sprinkled in the maze, mice were able to explore the maze and eat the cheese pieces freely for 5 min. On D6 and D7, cheese pieces were sprinkled only in the eight arms. The test stage started from D8 to D14, twice a day with an interval of 4 h. A piece of cheese with an approximate diameter of 1 mm was put in the end of each arm. The trial ended once mice finished all the food or past of 25 min. The right choice was defined as mice went into a new arm and took the cheese in the first eight entrances, the wrong choice was defined as mice re-entered an arm that they had already entered with no cheese in the end of the arm. Performances of mice were video recorded and the order in which mice entered each arm was recorded manually. The frequency of right and wrong choices was then calculated. Mice were fasted 4 h before each trial to ensure that the mice were starved during the test. The maze was wiped by 70% alcohol each time before mice start a new trial to exclude the odor effect. Mice were handled for 5 min a day before behavioral tests to avoid emotional affection.

### Object location recognition and novel object recognition test

Object location recognition test was performed to evaluate spatial working memory, based on the explorative innate of mice, as previously described [17, 18]. The experiment started on D28 after the operation, mice were put in animal behavior box with spatial cues marked on three sides of the wall for 3 d, each day 20 min to adapt to the environment. On D4, in the baseline session, two identical objects were placed on one side of the box, mice were allowed to explore freely for 10 min, in the test session, one of the objects was placed on the same location (familiar location), the other object was placed on the opposite end of the box (novel location). Mice were then put in the box to explore for another 10 min, the retention internal was 10 min as well. Performances of the mice were video recorded, orientation to the object within 2 cm, as well as interaction with the object (climbing, sniffing) were defined as exploration. The frequency and time spent exploring the familiar location (F) and novel location (N) were calculated by software. Discrimination index (N − F/N + F) was calculated for comparison. Discrimination index of exploring frequency was defined as the different value of the frequency exploring the novel and familiar location divided by the total exploring frequency. Discrimination index of exploring time was defined as the different value of time spent exploring the novel and familiar location divided by the total exploring time. Mice that have a preference for the two objects in the baseline session were excluded, box and objects were wiped by 70% alcohol once mice were out of the box to exclude the odor effect. Mice were handled for 5 min a day before behavioral tests to avoid emotional affection.

In the novel object recognition test, the adaption process was the same as object location recognition test. On D4, in the baseline session, objects A and B were put in the animal behavior box, mice were allowed to explore freely for 10 min, in the test session, object B was replaced by object C, mice were then put in the box to explore for another 10 min, the retention interval of the two sessions was 10 min as well. The frequency and time spent exploring object C and object A were analyzed. Discrimination index was calculated (C − A/C + A) for intergroup comparison, as described in the object location recognition test.

### Immunofluorescence staining

Mice were sacrificed 42 d after the operation for immunofluorescence staining. An overdose of pentobarbital sodium was injected intraperitoneally for anesthetization, the brains were removed, fixed with 4% paraformaldehyde, dehydrated with 20% and 30% sucrose. Brain slices with a thickness of 30 μm were cut by cryostat (Leica 1900, Germany). The sections were blocked in 5% BSA (Amresco, the United States) for 1 h at room temperature, then incubated with goat anti-Iba-1 (1:500 dilution, Abcam, United Kingdom) primary antibody at 4 ℃ for overnight, followed by AlexaFluor647 (1:500 dilution, Abcam, United Kingdom) secondary antibody for 1 h at room temperature. DAPI (1:2,000 dilution, Sigma, United States) was added in the mounting medium for labeling of cell nuclei. The sections were observed with confocal microscope (Olympus FV1000, Japan). Images were acquired with a 40 × objective and 800 × 800 pixel resolution. A 3D view with a depth of 10 μm was used for the calculation of Iba-1. The numbers of immunofluorescence positive cells were counted by an investigator who was blinded to different groups. Three sections per mouse were stained for statistical calculation.

### Assessment of Evans blue (EB) extravasation with spectrophotometer

BBB permeability was evaluated by EB, as previously described [19]. On D1, D3, D7, D42 after the operation, 2% EB (#E2129, sigma) was injected intraperitoneally and circulated for 2 h, mice were then anaesthetized with an overdose of pentobarbital sodium (1%, 10 ml/kg) intraperitoneally and then perfused with 37℃, 200 ml saline till the perfusing liquid turns clear. The brains were removed and cortical tissue was harvested and weighed. Tissue was grinded and formamide (1 ml/100 mg, Beijing tongguang fine chemical Co. LTD, China) were added, homogenized and then incubated at 60 ℃ for overnight to extract EB dye from the brain. The homogenate was centrifuged and the optical density of supernatants was measured at 620 nm by spectrophotometer (Thermo Scientific, United States). The cortical content of EB was expressed as the content of EB dye quantity in the brain (µg/g).

### Assessment of Evans Blue extravasation with confocal fluorescence microscope

Confocal fluorescence microscope (Olympus FV1000, Japan) was used to observe the EB extravasation more intuitively. On D1, D3, D7, D42 after the operation, 2% EB was injected intraperitoneally and circulated for 2 h. Mice were then anaesthetized by an overdose of pentobarbital sodium and perfused intracardially with 200 ml saline till the perfusing liquid turns clear and then followed by 50 ml 4% paraformaldehyde (PFA, pH 7.4). The brains were removed and post-fixed overnight in 4% PFA, then dehydrated with 20% and 30% sucrose at 4 °C. Brains were embedded in OCT (RuiTaibio, China), frozen at -80 °C, cut into coronal Sect. (20 μm) on cryostat (Leica 1900, Germany). EB was labeled as purple fluorescence and vessels were labeled as green fluorescence by confocal microscope. Total fluorescence intensities of EB extravasation were quantified by the NIH ImageJ 1.44 analysis software. 3 view fields per section, 3 non-adjacent sections each animal were chosen and average intensities were calculated. For statistical comparison, Average intensity of sham group was calculated and discrimination index were calculated as fold versus sham group.

Western blot analysis.

Mice were anaesthetized with an overdose of pentobarbital sodium (1%, 10 ml/kg) and then sacrificed by decapitation on D1, D3, D7, D42 after the operation. Cortical tissue was then dissected on ice, RIPA protein extract reagent (Applygen, China) containing protease inhibitor cocktail (Roche, Switzerland) was used for sample homogenization. Total proteins were abstracted and quantified by BCA method, boiled for 5 min and stored at -80 ℃ until further use. Homogenate aliquots (40 µg of total protein) were boiled for 5 min and then electrophoresed on SDS-PAGE acrylamide gels, transferred onto PVDF membrane (0.45 μm), blocked in Tris-buffered saline containing 0.05% Tween-20 and 5% nonfat milk (Applygen, China) for 1 h and then incubated with primary antibodies against β-actin (1:1000 dilution, Origene, China); ZO-1 (1:500 dilution, Invitrogen, United States); claudin-5 (1:1000 dilution, Abcam, United Kingdom); occludin (1:2000 dilution, Abcam, United Kingdom) at 4 °C for overnight. After washing with TBS-T, membranes were then incubated with peroxidase-conjugated anti-rabbit antibody (1:1,000 dilution, Origene, China); peroxidase-conjugated anti-mouse antibody (1:1,000 dilution, Origene, China) for 1 h at room temperature. Pre-stained protein ladders (#26,616, Thermo Scientific, United States) (#26,625, Thermo Scientific, United) were used as the molecular weight marker. Blots were assessed by an ECL system. Relative densities of the blots were semi-quantified with software Quantity One 4.6.

### Statistical analysis

Results were expressed as mean ± Standard Error of the Mean (SEM). Statistical comparisons were performed using GraphPad Prism software (version 7.04). Data was analyzed between the two groups by student’s t test. Two-way ANOVA with repeated analysis was conducted to compare differences between groups in radial arm maze test. Immunoblots were evaluated for statistical significance by two-way ANOVA followed by a multiple comparison test. Pearson correlation analysis was performed to assess the correlation between performance in radial arm maze test and BBB permeability. Differences in the values were considered significant at P < 0.05.

## Results

### BCAS mice showed working memory impairment in radial arm maze test

**Fig. 1:**
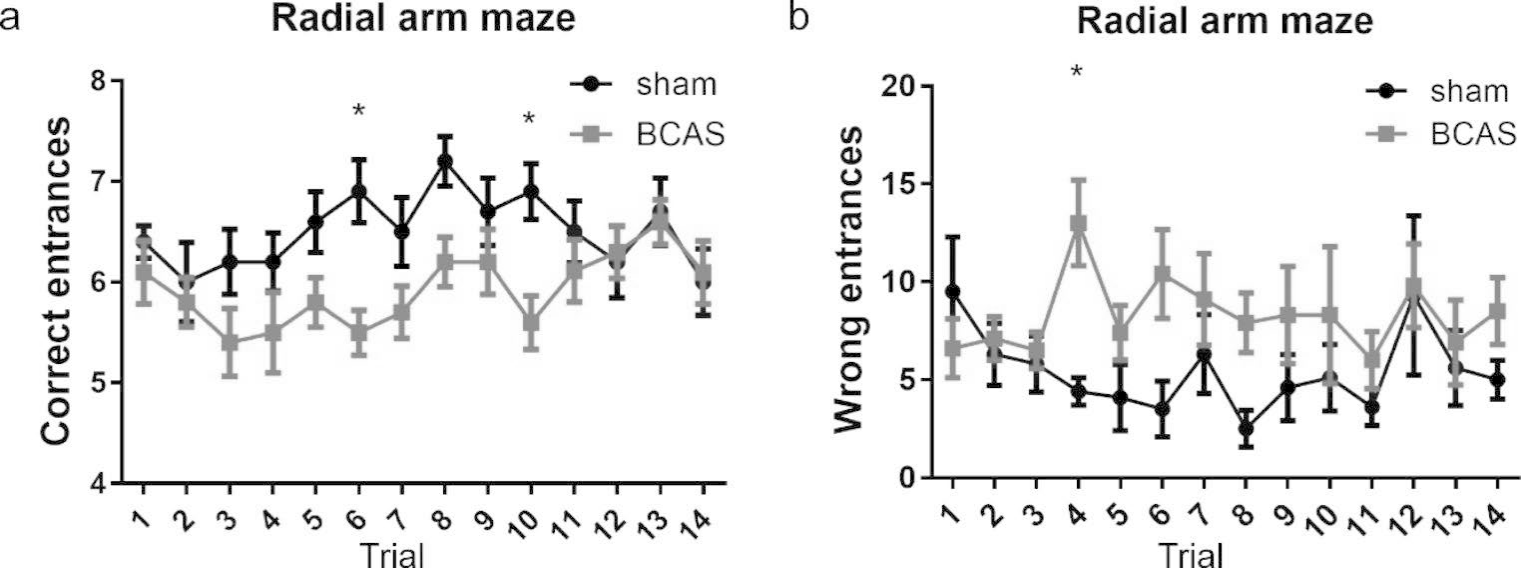
BCAS led to working memory impairment in radial arm maze test. BCAS and sham group showed significant difference in both correct entrances and wrong entrances (p < 0.05). BCAS mice showed significant decreased correct entrances in trial 6 and 10 (**a**) and increased wrong entrances in trial 4 (**b**) (n = 10, *p < 0.05 vs. sham group)

Young adult male *Tie2-GFP* mice were subjected to BCAS or sham operation. Radial arm maze test was performed to investigate working memory in BCAS and sham groups beginning on D28 after the operation. The test stage included 14 trials, twice a day with an interval of 4 h. Two-way ANOVA with repeated analysis was conducted to compare differences between groups. The two groups showed significant difference in both correct entrances and wrong entrances (p < 0.05). In the repeated analysis, less correct entrances in first eight entrances were shown in BCAS group (5.50 ± 0.22 in trial 6, 5.60 ± 0.27 in trial 10), compared with sham group (6.90 ± 0.31 in trial 6, 6.90 ± 0.28 in trial 10) (P < 0.05) (Fig. 1a), more wrong entrances were made to take all the cheese pieces or till the end of the test in BCAS group in trial 4 (13.00 ± 2.20), compared with sham group (4.40 ± 0.70) (P < 0.05) (Fig. 1b). The less correct and more wrong entrances revealed dysfunction of working memory in BCAS group. Altogether, BCAS mice showed working memory impairment compared with sham operation.

### BCAS mice showed impairments in object location recognition test and novel object recognition test

**Fig. 2:**
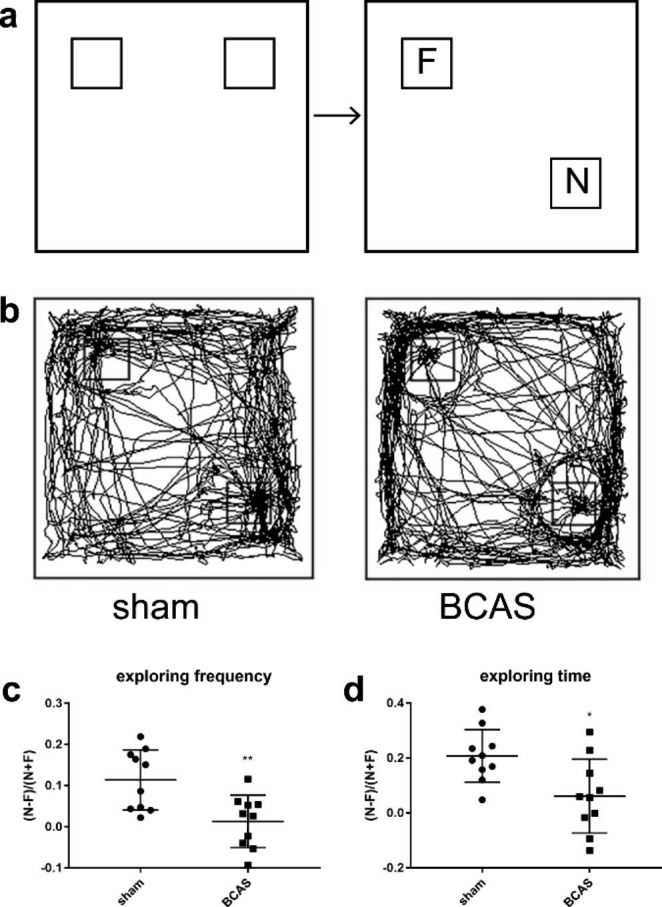
BCAS led to spatial memory impairment in object location recognition test. **a** Schematic diagram of object location recognition. **b** Representative tracks of sham and BCAS groups. **c, d** The exploring time and frequency decreased significantly in BCAS mice (n = 10, **p < 0.01 vs. sham group, *p < 0.05 vs. sham group)

**Fig. 3:**
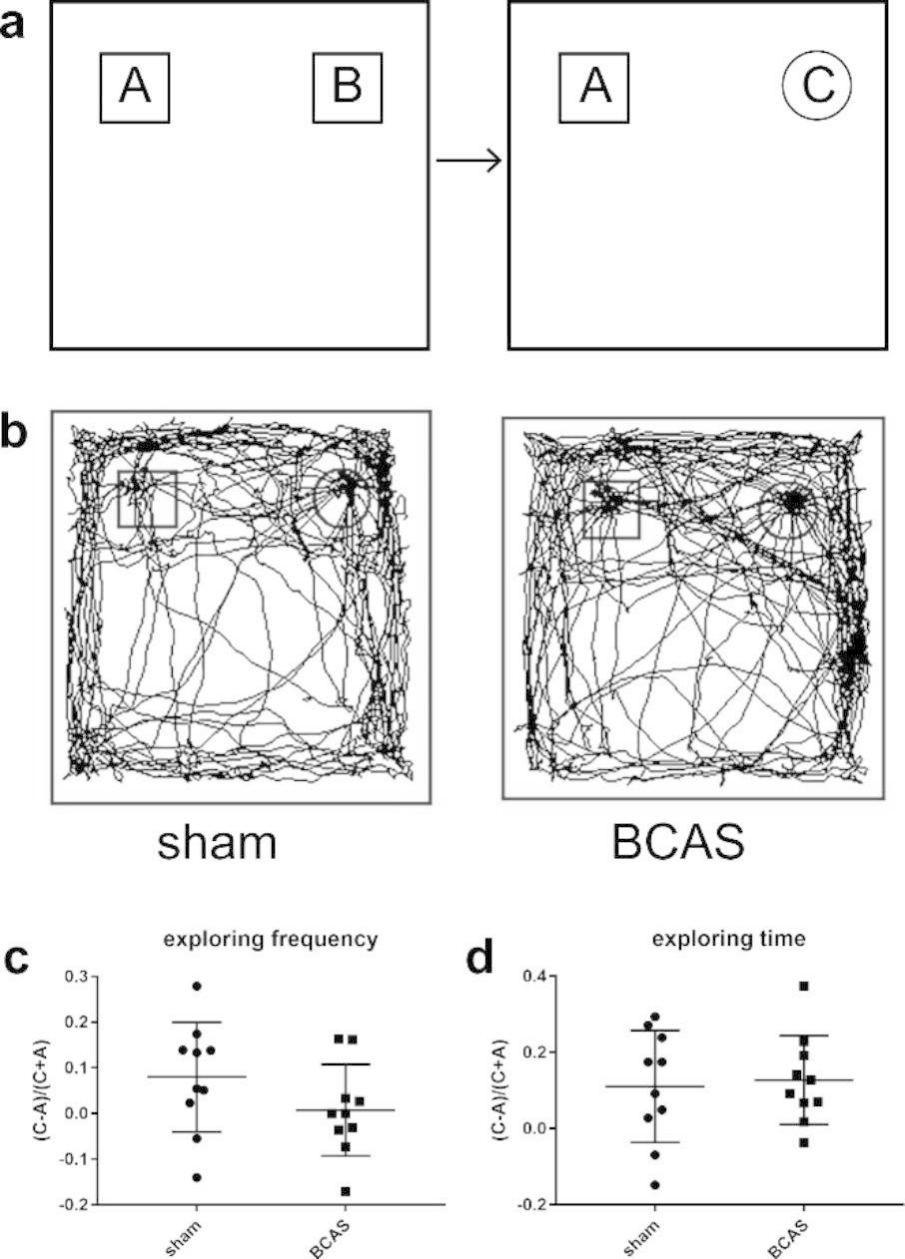
BCAS mice showed no significant differences with sham group in novel object recognition test. **a** Schematic diagram of novel object recognition test. **b** Representative tracks of sham and BCAS groups. **c, d** No significant differences were observed between the two groups (n = 10)

Object location recognition test and novel object recognition test were developed to assess spatially dependent memory in sham and BCAS groups beginning on D28 after the operation, based on the innate preference for novelty of mice. BCAS mice showed significantly less preference for novel location, evidenced by lower discrimination index of exploring frequency (P < 0.01) (Fig. 2c) and exploring time (P < 0.05) (Fig. 2d), compared with sham group in the test session. However, both discrimination index for exploring frequency and time showed no significant differences in BCAS and sham group in novel object recognition test (P > 0.05) (Fig. 3c, d). These results suggested that compared with sham operation, BCAS could lead to spatial memory impairments.

### BCAS mice exhibited increased quantity of microglia compared with sham mice

**Fig. 4:**
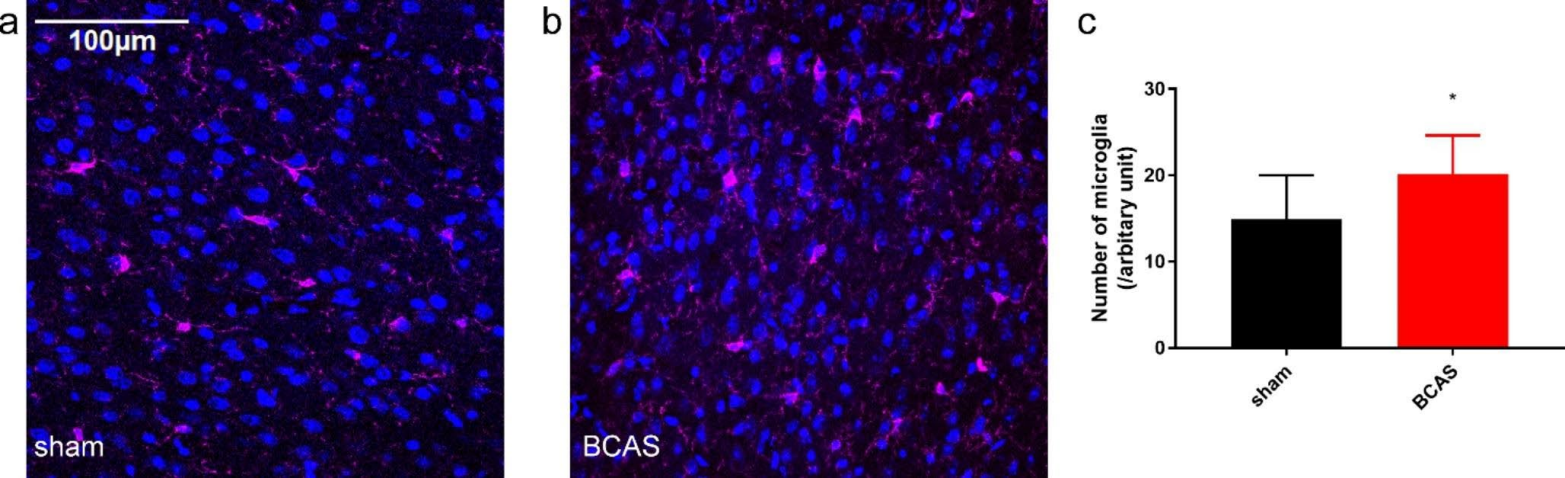
Representative microglia immunofluorescence labelled by Iba-1 in sham (**a**) and BCAS (**b**) groups. **c** BCAS mice showed increased microglia (n = 10, *p < 0.05, vs. sham group)

General marker ionized calcium-binding adapter molecule 1 (Iba-1) was used to label microglia. Mice were sacrificed 42 d after the operation and Goat anti-Iba-1 (Abcam, United Kingdom) antibody was used for immunofluorescence staining of microglia. Three sections in cerebral cortex per mouse were stained for analyzation. The numbers of immunofluorescence positive cells were counted. The normal distribution of the data was checked by KS normality test and the p value was > 0.10. Unpaired T test was used for the statistical analysis. On D42, BCAS mice exhibited increased quantity of microglia (20.15 ± 1.42), compared with sham group (14.94 ± 1.60) (P < 0.05) (Fig. 4a-c). These results indicated that cerebral hypoperfusion in this model led to an increase number of microglia.

### BBB Permeability was Increased in BCAS Group

**Fig. 5:**
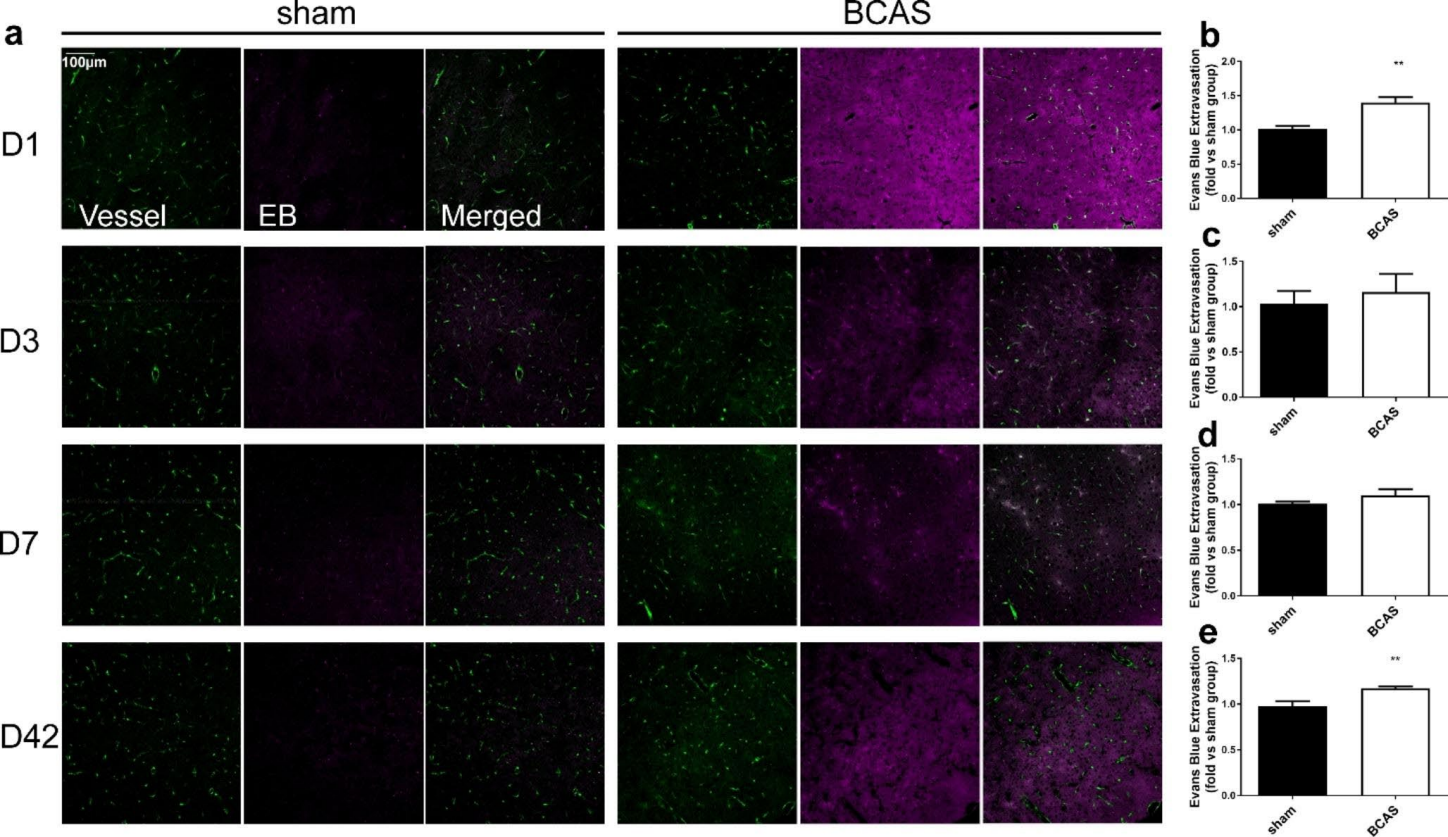
**a** representative EB fluorescence images in sham and BCAS groups. **b-e** EB total fluorescence intensities in sham and BCAS group on D1 (**b**), D3 (**c**), D7 (**d**) and D42 (**e**). Increased fluorescence intensities of EB were observed on D1 (**b**) and D42 (**e**) in BCAS group (n = 7, **p < 0.01 vs. sham group, measured by confocal microscope)

**Fig. 6:**
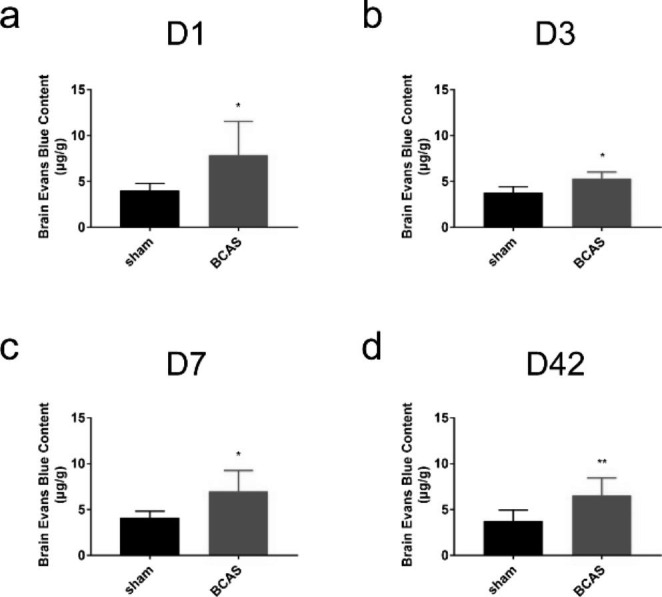
Brain EB dye content increased on D1 (**a**), D3 (**b**), D7 (**d**) and D42 (**d**) in BCAS mice. (n = 7, **p < 0.01 vs. sham group, *p < 0.05, vs. sham group, measured by spectrophotometer)

Evans blue extravasation was detected to evaluate BBB permeability. 2% EB was injected intraperitoneally on D1, D3, D7 and D42 after the BCAS or sham operation. EB was labeled as purple fluorescence and vessels were labeled as green fluorescence by confocal microscope. 3 view fields of cerebral cortex per section, 3 non-adjacent sections each animal were chosen and average intensities were analyzed. Distribution of EB around vessels or even the entire brain slices in BCAS group were observed on each time point, especially on D1, while little red fluorescence was detected in brain slices of the sham group (Fig. 5a). Analysis of fluorescence intensity suggested that EB extravasation was more than that in sham group on D1 (sham: 1.02 ± 0.04; BCAS: 1.40 ± 0.08) (P < 0.01) (Fig. 5b) and D42 (sham: 0.98 ± 0.05; BCAS: 1.17 ± 0.02) (P < 0.01) (Fig. 5e), slightly but not significantly more on D3 (sham: 1.04 ± 0.14; BCAS: 1.16 ± 0.20) (P > 0.05) (Fig. 5c) and D7 (sham: 1.01 ± 0.02; BCAS: 1.10 ± 0.07) (P > 0.05) (Fig. 5d). For further confirmation, we verified EB content at 620 nm using a spectrophotometer. Increased EB content was observed on each time point in BCAS group (D1: 7.65 ± 1.21; D3: 5.35 ± 0.29; D7: 6.75 ± 0.71; D42: 6.43 ± 0.66), compared with sham operation (D1: 4.12 ± 0.28; D3: 4.01 ± 0.35; D7: 4.46 ± 0.39; D42: 3.77 ± 0.44) (P < 0.05) (Fig. 6a-d). Taken together, our findings suggested that BBB permeability was increased in BCAS group, compared with sham operation.

### BCAS mice Showed Reduced Expression of TJs

**Fig. 7:**
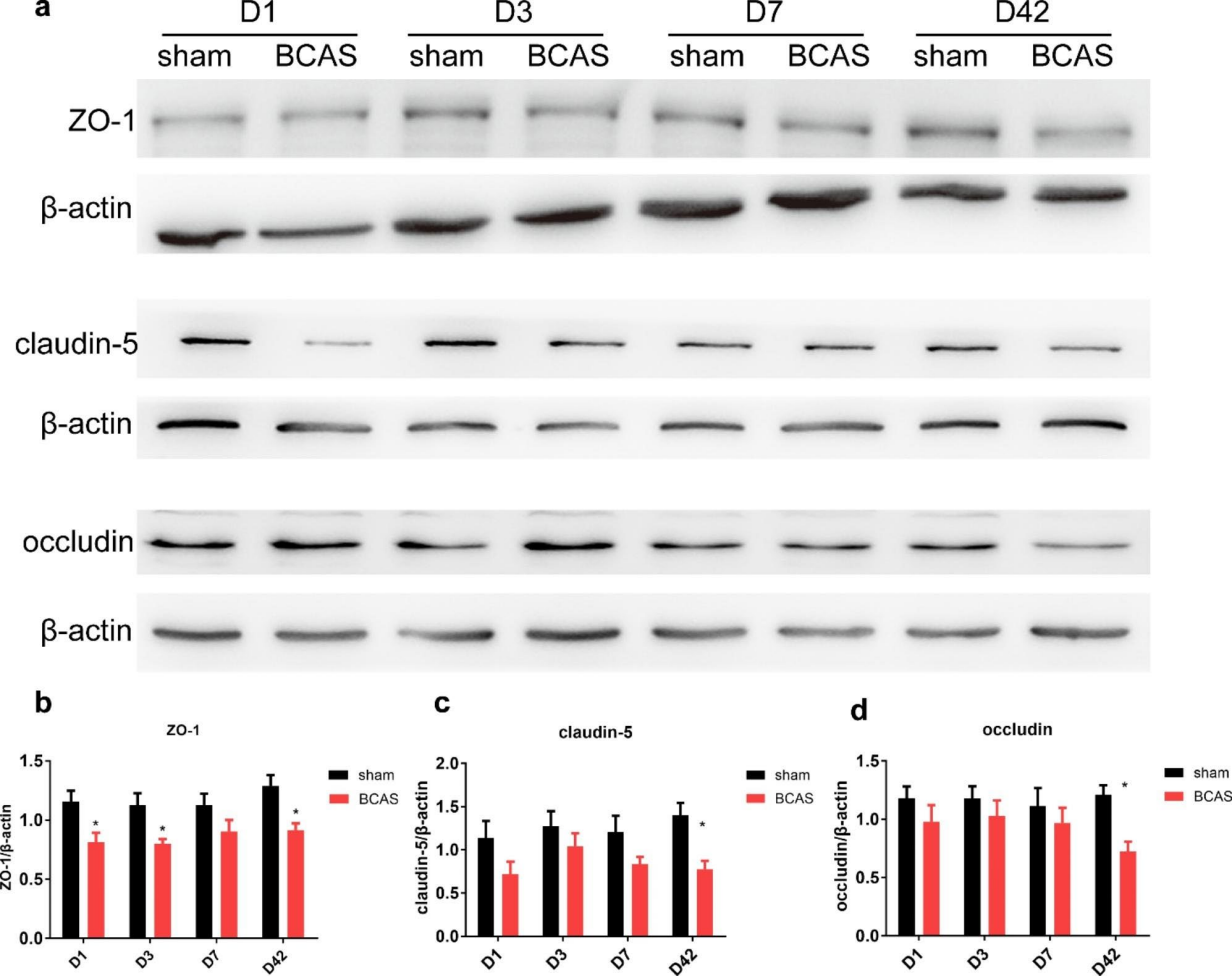
Western blot demonstrated decreased TJs expression in BCAS mice. **a** Representative immunoblots depicting the protein expression of tight junctions ZO-1, claudin-5 and occludin in both groups on D1, D3, D7 and D42 after the operation. **b-d** Densitometry analysis of the immunoblots (n = 7, *p < 0.05, vs. sham group)

To verify whether tight junction participated in changes of BBB permeability, we then evaluated expression of TJs ZO-1, claudin-5 and occludin by western blot analysis in brains of mice that subjected to BCAS or sham operation. Representative immunoblots showed decreased expression of ZO-1, claudin-5 and occludin in BCAS mice(Fig. 7a). Densitometry analysis of the immunoblots revealed that BCAS mice showed decreased ZO-1 expression on D1 (0.81 ± 0.08), D3 (0.78 ± 0.06) and D42 (0.92 ± 0.06) (P < 0.05) (Fig. 7b), but didn’t show significance on D7 (0.90 ± 0.10) compared with sham operation (D1: 1.16 ± 0.09; D3: 1.12 ± 0.10; D7: 1.13 ± 0.09; D42: 1.29 ± 0.09) (Fig. 7b). The expression of claudin-5 decreased significantly D42 in animals subjected to BCAS (0.78 ± 0.01) (P < 0.05) (Fig. 7c), slightly but not significantly decreased on D1 (0.72 ± 0.15), D3 (1.04 ± 0.15) and D7 (0.84 ± 0.08), compared with sham operation (D1: 1.14 ± 0.20; D3: 1.27 ± 0.18; D7: 1.21 ± 0.19; D42: 1.40 ± 0.14) (Fig. 7c). The expression of occludin decreased significantly on D42 in BCAS group (0.72 ± 0.08), compared with sham group (1.21 ± 0.08) (P < 0.05) (Fig. 7d), slightly but not significantly decreased on other time points (D1: 0.98 ± 0.14; D3: 1.03 ± 0.13; D7: 0.97 ± 0.13), compared with sham group (D1: 1.18 ± 0.10; D3: 1.18 ± 0.11; D7: 1.12 ± 0.15) (Fig. 7d). These results indicated that degradation of TJs could be induced by BCAS.

### Correlation Between Cognitive Performance and BBB Permeability

**Fig. 8:**
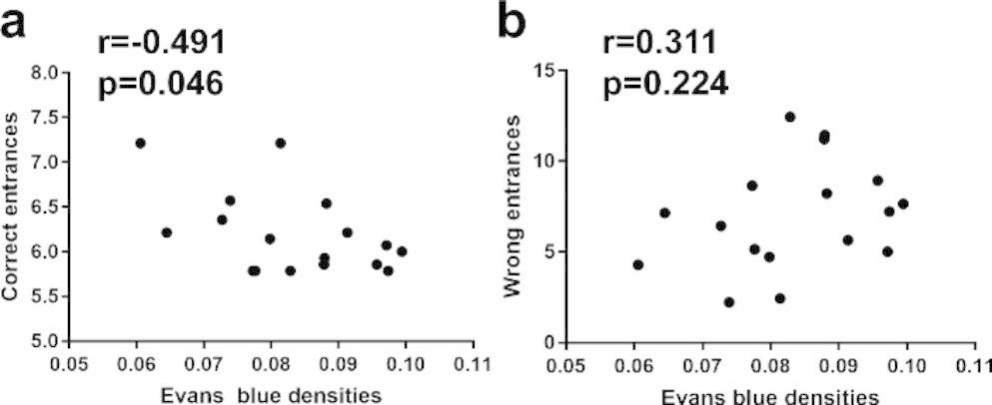
Correlations between cognitive performances and BBB permeability. **a** Correlation between mice correct entrances in radial arm maze test and EB extravasation. **b** Correlation between mice wrong entrances in radial arm maze test and EB extravasation (n = 17)

We assessed EB extravasation on mice that have just completed radial arm maze test. Pearson correlation analysis was used to verify whether there was a correlation between mice cognitive performance and BBB permeability. Right entrances and wrong entrances in the radial arm maze test were analyzed for cognitive performance, EB average extravasation per mice was analyzed for BBB permeability. Our results showed that correct entrances of mice in radial arm maze test had a moderate negative correlation with EB extravasation (r=-0.49, *p* < 0.05) (Fig. 8a), no significant correlation was found between wrong entrances and EB extravasation (Fig. 8b).

## Discussion

BCAS model as one of the best models for ischemic vascular dementia, was used in this study. The survival rate of this model fluctuated between 87.5% and 100%. BCAS surgery did not cause any mobility deficiencies in mice. Our data indicated that procedure of BCAS led to working memory and spatial memory impairments, accompanied with increased microglia. Moreover, increased BBB permeability on each time point was observed in BCAS mice. Additionally, reduced TJs expression in BCAS group after the operation was demonstrated. A moderate negative correlation between the cognitive performance in radial arm maze test and BBB permeability was found in this model.

To target the similar declining memory process as seen in humans with dementia, several tasks were developed to assess rodent memory acquisition, as previously described, with water maze test to assess spatial memory acquisition and some working memory, radial arm maze test to assess spatial and working reference memory, novel object recognition test to assess associative memory and declarative as well as working memory, object location recognition test as a modification of novel object recognition to assess working memory and spatial memory, fear conditioning memory test to assess associative memory, and Y maze to assess active working short term memory [20]. In our present study, radial arm maze test, object location recognition and novel location recognition test were implemented to evaluate cognition, so as to cover as many cognition types as possible. Water maze test were not conducted since our previous study has demonstrated that mice subjected to BCAS operation suffer cognitive deficits compared with sham group in the test [15], so as to reduce the stress and pain that could be caused during the tests.

In radial arm maze test, BCAS mice exhibited significantly less correct entrances and more wrong entrances compared with sham mice. This result was consistent with previous studies [21], indicating that CCH induced spatial memory impairment [22]. Object location recognition test was developed utilizing rodents’ natural exploratory process to assess working memory as well as spatial memory demanding for less cost and time, compared with other behavioral tests. Here, in object location recognition test, BCAS mice showed less exploring preference for novel location. Since novel location recognition assesses more spatially dependent memory [18], the results suggested that BCAS mice suffered more spatial memory deficits compared with sham mice. The two groups didn’t show significant differences in novel object recognition test. However, study previously reported by Patel A showed that BCAS mice exhibited significant deficits in short-term memory [23]. One possible explanation accounting for this contradiction is that since manual analysis was imprecise in the record of exploring time and might also exist experimenter bias, as was used in the previous study, the software systems for scoring exploration we used reduced artificial error but also brought about a question that the software was not able to make discriminations when mice occasionally sit on or near the object without actively exploring the object. Further confirmation should be implemented. These results indicate that BCAS procedure could lead to cognitive impairments.

Neuroinflammation was found playing dual roles in response to brain ischemia [24] and involved in many other brain diseases [25]. Microglia are the main resident immune cells in the brain fulfilling protective functions in the central nervous system. They could also produce and release proinflammatory factors and inflammatory mediators, which in turn activate more astrocytes and microglia, and promote the release of more inflammatory mediums in the pathological state. This feedback loop propels neuroinflammation that results in brain injury [26]. Therefore, microglia are the first line defending harmful stimulations [27]. Studies have suggested that microglia play vital roles in development and homeostasis of the central nervous system [28]. Activation of microglia was proved to have crosstalk with the p38 MAPK-NF-κB signaling pathway, in which MMP9, as an inflammatory mediator but also a metalloprotease constituting BBB, is involved. Activated microglia presents functional dichotomy, M1 polarization exerts proinflammatory and neurotoxic effects, while M2 polarization exerts anti-inflammatory effects [29]. Microglia are demonstrated to produce neuroregulatory factors that support synaptic plasticity [30], white matter injury in multiple sclerosis is accompanied by BBB dysfunction and glial activation, which is characterized by marked morphological changes [30]. In the present study, increased microglia were demonstrated in the group that suffered BCAS procedure. Since we could not distinguish M1-type (classical/proinflammation activation) and M2-type (alternative/anti-inflammatory activation) simply by cellular morphology analysis, which play beneficial or detrimental roles [29], double labelling of Iba-1 with M1 marker and M2 marker is an exciting work to further explore the role of microglia in CCH in our future research. BBB disruption occurs in early phase of many central nerves system diseases, such as subarachnoid hemorrhage [31], multiple sclerosis [32], stroke [33], and Alzheimer’s disease [34] Various components of the blood-brain barrier change when vascular events occur, leading to dysfunction and increased permeability, and may ultimately causing cognitive impairment [12]. Endodermis of cerebral vessels could generate Nitric Oxide, which participated in cerebral blood flow regulation and cognitive processes [39]. It has been demonstrated that L-arginine improved the utilization of NO in the brain, thereby ameliorated learning and memory function [40]. Inverse relationship was found between pericyte coverage and blood-brain barrier permeability [41]. The phagocytic properties of pericytes help to remove toxic circulating plasma proteins such as immunoglobulin and albumin from brain tissue [41]. In addition, specific receptor proteins expressed by pericytes could clear macromolecules such as Aβ and lipoprotein receptor-associated protein 1 [42]. Therefore, pericyte degeneration may lead to increased BBB permeability and accumulation of metabolites, which may participate in the vascular pathology of AD. Astrogliosis developed in the damaged area under condition of ischemia and hypoxia, enhancing neuronal plasticity and promoting regeneration [43]. Nevertheless, overactivation of astrocytes induced glial scarring and inhibited axonal regeneration [44]. ET-1 increased due to astrocyte activation under ischemic conditions and bound to its receptor RAGE, which was expressed by astrocyte, by prompting the cleavage of amyloid precursor protein (APP), thus could produce more Aβ [45]. Protection of BBB is demonstrated involved in attenuation of cognitive impairment [46]. c The role of BBB in cognitive impairments caused by CCH still needs further exploration.

In the present study, we evaluated BBB permeability in cerebral cortex on D1, D3, D7 and D42 after BCAS procedure by two ways using EB. EB is a widely used marker to evaluate brain barrier integrity [47]. Our results showed higher fluorescence intensity on brain slices in BCAS mice on D1 and D42, on D3 and D7 after the operation, the fluorescence in BCAS group tended to be higher but showed no significance, compared with sham group. In addition, we evaluated brain EB content at 620 nm by spectrophotometer. Increased EB content was observed on each time point in BCAS group, compared with sham operation. Comparable to previous studies which most focused on the early stage after the BCAS procedure [48], our results also suggested that increased BBB permeability could occur throughout the BCAS procedure. These results are slightly different from study reported previously that BBB dysfunction was most severe 3 days after the BCAS, and recovered gradually during the following 30 days in the cortex, though Evans blue integrate densities in BCAS mice were still significantly higher than sham group on D30 [49], the reason of this difference may be that the mice we used in this study were not *C57BL/6* mice, the degree of anastomosis between carotid and vertebrobasilar circulation at the circle of Willis was different between strains [50], besides, we did not compare BBB permeability between different time points, since we performed the surgery at a relatively strict week old and body weight, yet the mice body weight was still increasing over time, we couldn’t exclude the influence of body weight and age.

TJs are key components sealing the gap between adjacent endothelial cells, forming a continuous, impermeable membrane that constituted the primary anatomical substrate of the BBB [51]. Main TJs include claudins, occludin and membrane-associated guanylate kinase-like proteins, ZO-1 for instance [52]. Decrease in TJs expression was extensively reported after brain ischemia [53, 54]. In the present study, the expression of TJs ZO-1, claudin-5 and occludin was evaluated by western blot analysis in cerebral cortex on D1, D3, D7 and D42 after the BCAS procedure. The results demonstrated significantly decreased expression of ZO-1 on D1, D3 and D42 in BCAS group, and decreased tendency on D7. BCAS group showed significantly decreased expression of claudin-5 and occluding on D42. Though gentle operation was implemented, mice still suffered carotid artery stenosis in a short time, this might explain the early decrease of TJs after BCAS. Nevertheless, it is worth mentioning that TJs remained decreased on D42 after the BCAS, this might be one reason responsible for the cognitive impairment. Observation on tight junction protein distribution changes through immunohistochemistry should also be conducted in our future study.

In this study, we evaluated longer term changes in blood-brain barrier, we found that BBB permeability was still increased in D42. Besides, we tried to figure out whether there was a relationship between BBB changes and cognition in this BCAS model, we performed correlation analysis between Evans blue extravasation and mice performance in radial arm maze test and demonstrated that Evans blue extravasation had a significant moderate negative correlation with mice correct entrances in radial arm maze test (p < 0.05), indicating that permeability of BBB may be involved in the cognitive changes induced by CCH. We are also willing to try pharmacological modulation of BBB permeability to further clarify whether it could lead to reduced cognitive deficits in the future. We hope these results could provide a reference for future interventions to improve cognitive impairments caused by chronic cerebral hypoperfusion.

There are limitations of this study. This model could not exactly mimic the real situation in patients suffering CCH, since the narrow progress gradually rather than abrupt stenosis in the model. Besidesc, we did not verify the changes of other components forming BBB or TJs distribution. In addition, how BBB plays its role exactly in cognitive changes after BCAS still needs further confirmation.

## Data Availability

All data included in this study are available upon request by contact with the corresponding author.
